# Beyond Turing: mechanochemical pattern formation in biological tissues

**DOI:** 10.1186/s13062-016-0124-7

**Published:** 2016-05-04

**Authors:** Moritz Mercker, Felix Brinkmann, Anna Marciniak-Czochra, Thomas Richter

**Affiliations:** Institute of Applied Mathematics, BioQuant and Interdisciplinary Center of Scientific Computing (IWR), Heidelberg University, Heidelberg, Germany; Department Mathematik, FAU Erlangen-Nürnberg, Erlangen, Germany

**Keywords:** Morphogens, Tissue morphogenesis, Development, Pattern formation, Mechanochemistry, Tissue mechanics, Mechanotransduction, Reaction-diffusion, Long-range inhibition

## Abstract

**Background:**

During embryogenesis, chemical (morphogen) and mechanical patterns develop within tissues in a self-organized way. More than 60 years ago, Turing proposed his famous reaction-diffusion model for such processes, assuming chemical interactions as the main driving force in tissue patterning. However, experimental identification of corresponding molecular candidates is still incomplete. Recent results suggest that beside morphogens, also tissue mechanics play a significant role in these patterning processes.

**Results:**

Combining continuous finite strain with discrete cellular tissue models, we present and numerically investigate mechanochemical processes, in which morphogen dynamics and tissue mechanics are coupled by feedback loops. We consider three different mechanical cues involved in such feedbacks: strain, stress, and compression. Based on experimental results, for each case, we present a feedback loop spontaneously creating robust mechanochemical patterns. In contrast to Turing-type models, simple mechanochemical interaction terms are sufficient to create *de novo* patterns.

**Conclusions:**

Our results emphasize mechanochemical processes as possible candidates controlling different steps of embryogenesis. To motivate further experimental research discovering related mechanisms in living tissues, we also present predictive *in silicio* experiments.

**Reviewers:**

Reviewer 1 - Marek Kimmel; Reviewer 2 - Konstantin Doubrovinski (nominated by Ned Wingreen); Reviewer 3 - Jun Allard (nominated by William Hlavacek).

## Background

During embryonic development, a fertilized cell develops step by step into a complex-patterned and shaped organism [15]. The mechanisms underlying these self-organized patterning processes have been the focus of research since several decades. The first relevant model goes back to the seminal work of Alan Turing [53]. It is based on the observation that interactions between two diffusing chemical species (termed ”morphogens”) with significantly different diffusion rates may lead to symmetry breaking and formation of periodic patterns. The most famous realization of the Turing’s idea is the activator-inhibitor model proposed by Gierer and Meinhardt [14]. Its basic version is given by two coupled reaction-diffusion equations [25]:

$$\begin{array}{@{}rcl@{}} \partial_{t} c &=& \rho_{c} \left(c^{2} /h - c\right) + d_{c} \Delta c,\\ \partial_{t} h &=& \rho_{h} \left(c^{2} - h\right) + d_{h} \Delta h, \end{array} $$

with zero-flux boundary conditions. Variables *c* and *h* denote concentrations of two morphogens, called activator and inhibitor, respectively. The parameters *ρ*_*c*_ and *ρ*_*h*_ are production/removal rates, and *d*_*c*_ and *d*_*h*_ denote the diffusion coefficients.

Mathematical analysis of the equations provides explanation for the phenomenon postulated by Turing, for details see [33, 39]. The mechanism is related to a local behavior of solutions of a reaction-diffusion system in the neighborhood of a constant stationary solution that is destabilized through diffusion. Patterns arise through a bifurcation, called diffusion-driven instability (DDI). The specific choice of model kinetics and distinct different diffusion rates *d*_*h*_>>*d*_*c*_ are necessary to induce the DDI (c.f., Fig. 5a). Consequently, after more than 60 years of research, molecular identification of the Turing-type molecules during morphological events remains an exception rather than a rule [16, 26]. Especially, appropriate candidates for fast diffusing long range inhibitors are usually missing. Interestingly, recent results suggest that in general the principle of “long-range inhibition/short range activation” holds. However, specific realizations seem to be versatile: e.g., primarily mechanical mechanisms have been shown to produce periodic tissue patterns as well (reviewed in [16]). Mechanical cues such as traction, rigidity, or compression underlie functions in patterning [7, 24, 40, 51], which have been ascribed to diffusing morphogens within Turing-type models. The active role of tissue mechanics in tissue patterning becomes justified by the fact that an increasing amount of data shows how gene expression and signaling cascades sense and process mechanical cues (for reviews, c.f. [5, 6, 12, 19, 30–32, 41, 44, 56, 57]). Taken together, these data suggest that a close interplay between chemical and mechanical processes within tissues may lead to pattern formation in many cases.

Despite the large number of experimental data, modeling approaches explaining *de novo* pattern formation as results of mechanochemical processes are still rare. Existing models integrate the tissue mechanics only in a simplified way [36, 37, 52]. Thus, the need for new modeling approaches has been recently stressed [18, 55]. A general challenge in such mechanochemical models is the description of the mechanical part of possible mechanisms, since it is non-intuitive from a modeling/numerical point of view. Already Turing suggested in his seminal paper the consideration of mechanical aspects in pattern formation, but restricted his own study to the chemical processes, since *”...the interdependence of the chemical and mechanical data adds enormously to the difficulty”* [53]. Currently, modeling and computation approaches describing mechanical aspects of morphogenesis have reached an advanced level (for reviews, c.f. [48, 58]), making the new models feasible.

In the current study, we propose and investigate integrated mechanochemical models to investigate tissue pattern formation. The models are based on the finite strain theory [17] and are extensions of the works as presented in [1]. Based on experimental data, we model different mechanochemical feedback loops by coupling diffusing morphogens with different mechanical cues, such as compression/stretch [5], strain/cell-shape [29, 56] or stress [28, 41, 42]. We numerically investigate resulting equilibrium patterns and their dependence on diffusion rates, initial conditions or the system size. Finally, we propose experimental approaches to decisively test analogous mechanochemical interplays in vivo.

## Results and discussion

In this section, we present and discuss several simulation results which are based on the proposed mechanochemical tissue models. In particularly, we combine continuum-based finite strain models with discrete models for biological cells. Furthermore, the simulated tissue geometry is based on an annulus geometry, so that we consider a thin loop of tissue confined to the plane. For more details regarding the model geometry, the modeling and simulation approach, the underlying biological/biophysical assumptions, as well as the detailed parameter setup, we refer to the “Methods” section.

### Mechanochemical pattern formation

**Fig. 1:**
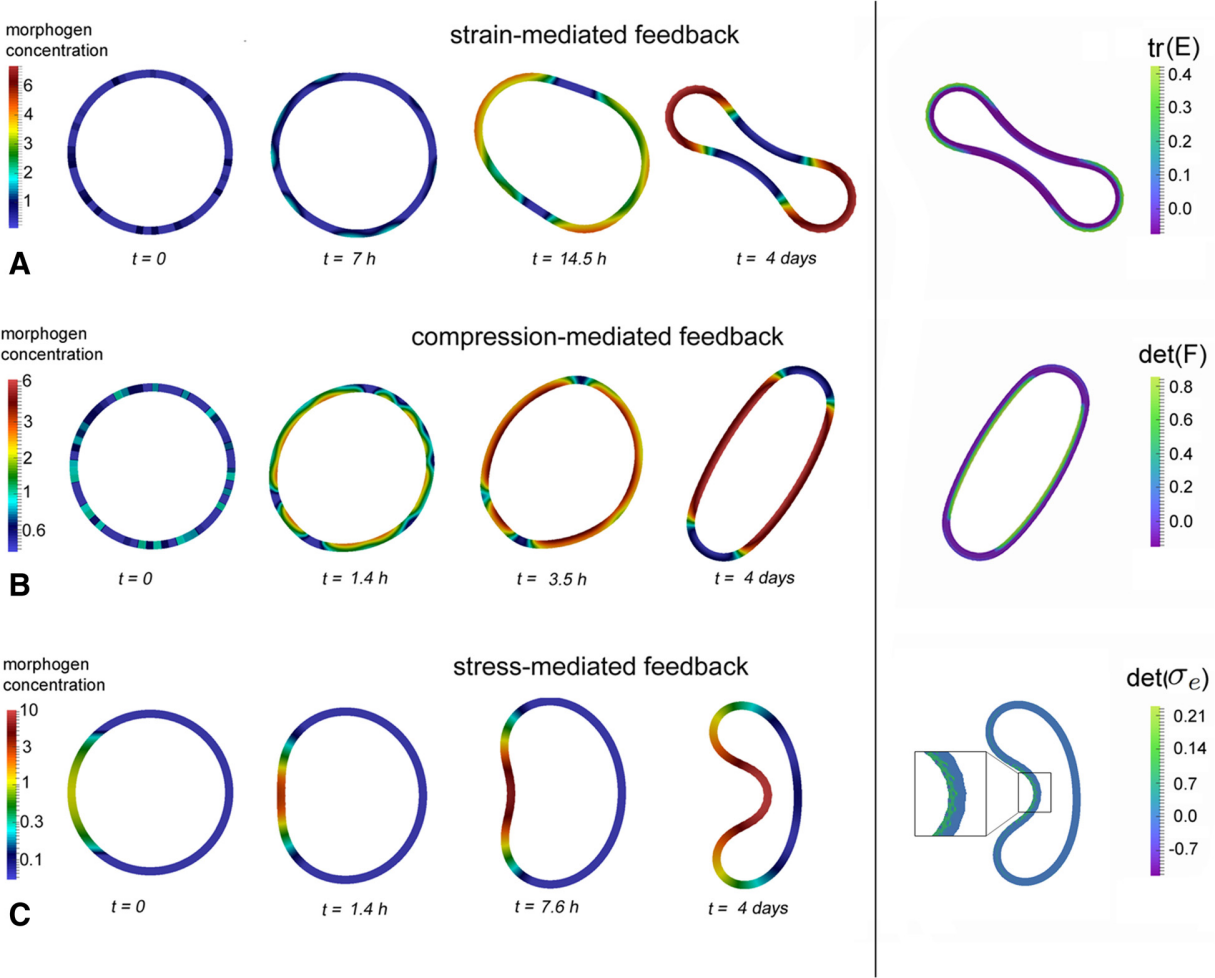
Simulation snapshots of tissue pattern formation based on different mechanochemical feedback loops. **a** Strain-mediated feedback-loop, starting from stochastic initial conditions; **b** Compression-mediated feedback loop, starting from stochastic initial conditions; **c** Stress-mediated feedback loop, starting from a ”maternal gradient” as a chemical pre-pattern. Heat maps on the *left-hand side* show morphogen concentrations, heat maps on the *right-hand side* show mechanical invariants used within the corresponding feedback-loop

In the simulation studies, we unraveled several different feedback loops between mechanical cues and morphogen dynamics, spontaneously leading to mechanical and chemical pattern formation. In Fig. 1, simulation results of three feedbacks based on strain (Fig. 1a), compression (Fig. 1b), and stress (Fig. 1c) are shown. Starting with either stochastically distributed morphogen (Fig. 1a, b) or a prescribed morphogen gradient (Fig. 1c) within an undeformed tissue loop, each feedback loop develops symmetric and stable curvature and morphogen patterns. Both types of initial conditions frequently appear in tissue morphogenesis. For example, in *Drosophila* development a prescribed maternal gradient determines the orientation of the body axis [20]. Other systems, from lower animals to organs of mammals, can regenerate spontaneously from aggregates of randomly mixed cells [13, 21], and thus have the capacity of *de novo* patterning.

We want to point out that the ability to form patterns in all three mechanochemical feedback loops does not critically depend on the initial conditions: Each presented feedback loop yields spontaneous emergence of stable patterns starting from a stochastically as well as a gradually distributed morphogen (results not shown).

In all presented simulations, we consider an apical or a basal constriction as an active part of the deformation, since such deformation processes appear to be frequently involved in tissue morphogenesis [34, 38, 50]. However, other possible active processes – such as isotropic or anisotropic tissue growth – can also play an important role in morphogenesis, especially in embryogenesis [2, 10], and are not considered in the models presented. Although detailed results are postponed to future works, our first simulation studies indicate that also here, different mechanochemical feedback loops spontaneously create robust patterns (results not shown). Thus, mechanochemical pattern formation does not critically depend on the exact nature of the active deformation.

Our simulation results furthermore reveal that the resulting equilibrium patterns can qualitatively differ, depending among others on the specific type of the mechanical cue involved in the feedback. Most frequent patterns show at least two morphogen/deformation patches arranged in a symmetric manner, such as foot-sole shapes (Fig. 1a, right) or ellipses (Fig. 1b, right), which can be generated by each of the three feedback loops. But also patterns with only one morphogen/curvature patch are possible (Fig. 1c, right), resembling those appearing during gastrulation event in embryogenesis [23]. Interestingly, the latter geometry we only observed in the case of the stress-mediated feedback loop, and starting with a gradually distributed morphogen. Thus, the symmetry and type of final patterns may critically depend on initial conditions and the type of the mechanochemical feedback loop.

It is worth mentioning that the patterns resulting from simulation of tissues restricted to the 2D plane may distinctly differ from those of full 3D tissues, for several reasons: From the mechanical point of view, we assume that the 3D nature of a blastula coupling cells and introduction of additional curvature may affect model outcomes. From the chemical point of view, a process such as circumferential pattern formation lacks one dimension, which implies that no distinction is made between stripes and spots. Thus, the models investigated in this work can be treated as test cases showing that a variety of stable patterns is produced from mechanochemical feedback loops, while for a comparison with experimentally observed structures 3D models are needed. We postpone the extension to the 3D models to future research.

### Mechanics versus diffusion in pattern formation

We emphasize the crucial role of tissue mechanics in short-range activation and long-range inhibition in all feedback loops considered: If cells are locally constricted due to increased local morphogen abundance, two simultaneous events promote subsequent pattern formation, 1) the local deformations induce an increased morphogen production at the same place, which leads to even stronger deformations and as a result we obtain a short-range activation of the morphogen; 2) the local deformations immediately lead to stress/strain/compression at surrounding tissue areas, which inhibits the local production of the morphogen at these places. As a result, in this latter case we obtain a mechanically mediated long-range inhibition, and local high morphogen concentration patches can inhibit other patches over long ranges.

**Fig. 2:**
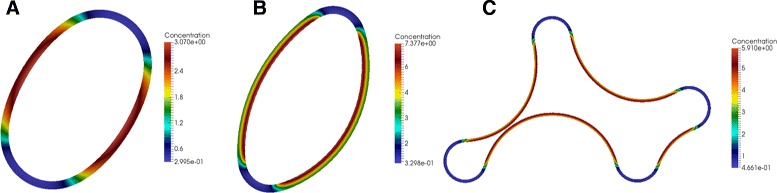
Equilibrium patterns resulting from the compression-mediated feedback loop. **a**, **b** Increasing the diffusion does not change the number of morphogen/curvature patches (**a** shows 10 times more diffusion compared to **b**). **c** In contrast, changing the mechanical properties of the system e.g. by quartering the tissue thickness effects the final number of morphogen patches

In contrast to tissue mechanics, diffusing morphogens play a mediating and thus subordinate role in patterning. The dominant role of mechanics becomes obvious by considering for example the spatial scaling of the final patterns, e.g., using the example of the compression-mediated feedback (Fig. 2). Strong differences in diffusion rates (Fig. 2 A results from 10 times more diffusion compared to Fig. 2b) do not change the final number of morphogen patches. In contrast, the number of patches scales with the tissue thickness. As a result, with one-quarter of the tissue thickness but the same diffusion rate as in Fig. 2a, we obtain four morphogen patches instead of two (Fig. 2c). However, we observe similar effects of tissue thickness and diffusion on the scaling of final patterns using the strain-mediated feedback instead (results not shown). We thus assume that these findings are robust to changes in the mechanochemical feedback loop.

### Predictive experiments

Experimental verification of mechanochemical feedback loops in vivo related to those presented in this study is a challenging task, and requires a joint manipulation and analysis of both, chemical and mechanical cues. While experiments including chemical (morphogen) manipulations and measurements are well established [46, 49], analogous techniques involving tissue mechanics are relatively young, but also reached an advanced level during recent years [48]. In the following, we propose and simulate possible techniques assessing the basic signaling structure within mechanochemical feedback loops. Such structure mainly consists of the two causal couplings, connecting mechanics with morphogen dynamics. We require

1. the existence of a morphogen locally changing tissue mechanics, as well as
2. a reverse signal from mechanics back to chemistry, e.g., a mechanical cue changing effective morphogen spread and/or production.

Having selected a certain morphogen candidate, each causality direction can be subsequently separately assessed: To prove (1), the morphogen of interest could be locally and ectopically over-expressed, with a subsequent search for the induction of colocalized mechanical patterns (such as deformations, c.f. [45]). Although the visualization of mechanical patterns different from deformations (such as stress or compression) is still challenging (e.g., [42]), recent technical developments are promising [22, 48]. Analogously, to assess item (2), the tissue can be actively deformed [11, 48], combined with a subsequent screen for accordingly aligned morphogen expression patterns.

**Fig. 3:**
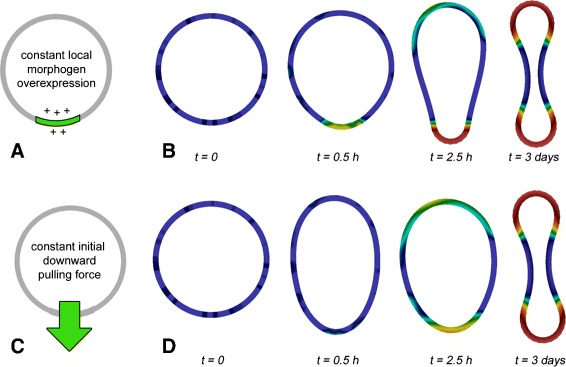
Two coupled predictive *in silicio* experiments for the detection of a mechanochemical feedback-loop in tissues. **a**, **b** Ectopic overexpression of the morphogen of interest leads to an alignment of resulting mechanical patterns relative to the chemical source. **c**, **d** An initial external mechanical deformation leads to the alignment of resulting morphogen patterns relative to the mechanical cue. **a**, **c** schematic view, **b**, **d** simulation results

To demonstrate a possible experimental outcome indicating the existence of a mechanochemical feedback, we have performed the above mentioned experiments virtually (“predictive *in silicio* experiments”) using the strain-mediated feedback loop as an example. Thus, the mechanical cue under investigation is a local tissue strain or bending. To mimic ectopic morphogen production, we have locally added a constant morphogen production term to the reaction-diffusion equation (schematic view: Fig. 3a). To simulate, in contrast, a prescribed external deformation at the same place (schematic view: Fig. 3c), we have added a local outward-pulling force to the structural equation (for more mathematical details, c.f. Section “Methods”). Starting with stochastically distributed morphogen, we observe in both cases that final mechanochemical patterns align to the source of constant morphogen production and pulling, respectively (Fig. 3b, d). Thus, comparable experimental readouts indicate a possible interplay between the morphogen of interest and tissue mechanics.

## Conclusions

To summarize, in this publication we have presented and investigated a class of models of pattern formation in biological tissues, namely mechanochemical models. We simulated three different simple feedback loops between mechanical cues and morphogen dynamics. In all three cases, we have shown that these type of interactions lead to a *de novo* formation of stable chemical and mechanical patterns. These results indicate that there are various possible ways in which a tissue may produce patterns by simple mechanochemical interplays. Furthermore, several of our predictions from simulations appear to be robust regarding changes in the mathematical model, such as changes in the type of the mechanochemical feedback loop (i.e., changes in the involved mechanical invariant or active deformations process), or in the exact choice of initial conditions.

Thus, mechanochemical patterning processes could be a likely candidate for the realistic and often still unknown processes underlying various developmental steps, for the following main reasons:

- In contrast to the Turing-type models, within presented feedback-loops, simple (linear) interaction terms and moderate diffusion rates are sufficient to produce robust *de novo* patterns. This makes the evolution of such mechanisms more likely.
- Within presented mechanisms, mainly mechanical cues undertake functions in short-range activation and long-range inhibition, chemicals (morphogens) play only a mediating and thus subordinate role. This could explain why the experimental identification of relevant molecular candidates is still missing in many cases.
- Especially in developmental steps where tissue deformations play an important role (e.g., during gastrulation), presented feedback loops display a natural and simple way for the tissue to control the progress and success of deformations, since the latter directly retroact on morphogen dynamics.

One of the main aims of this paper is to motivate further experimental research in order to validate mechanochemical mechanisms for tissue pattern formation. Recent experimental observations [4], theoretical results [37], as well as technical developments [22, 48] are however promising. If mechanochemical mechanisms are validated in tissues by direct experimentation (for example similar to the methods as suggested in Fig. 3) this will constitute an essential step in the understanding of embryogenesis - one of the greatest current mysteries in biology [23, 39].

## Methods

### Model geometry

**Fig. 4:**
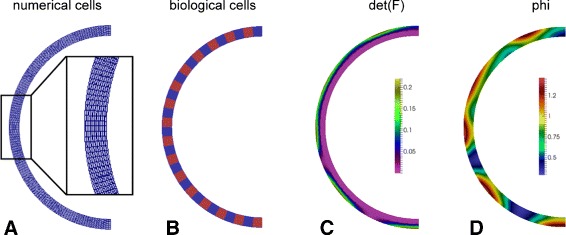
Section of a simulation considering an evolving tissue loop after 15 *m* *i* *n*, using the example of the compression-mediated feedback. **a** Finite element discretization, **b** biological cells, **c** mechanical cue (here tissue compression quantified by det(**F**)), **d** morphogen concentration *ϕ*

As an example, we investigate a system resembling the blastula stage of an embryo. Specifically, we parameterize over annulus geometry, which means that we consider a 2D cross section through a blastula, i.e. a tissue loop with a finite thickness which is confined to the 2D plane. This loop represents one cell layer which is composed of 64 circumferentially arranged biological cells (Fig. 4b). The outer radius of this tissue sphere constituting the outer border of the annulus is 150 *μm*, and the inner radius is 135 *μm*, resulting in tissue thickness of 15 *μm*.

### Combining continuous and discrete tissue models

The presented modeling approach combines continuous (finite strain) modeling techniques with a discrete model for the shape and position of biological cells. The explicit distinction of biological cells from numerical cells (finite elements) is necessary to appropriately describe active shape changes of biological cells, without being restricted by the local resolution of continuous mechanical processes determined by finite elements.

As an example, the active cell deformation processes of apical or basal constriction (c.f., following subsections) cannot be described within a purely continuous framework: although applied to the overall body, active deformations (given by **F**_*a*_, c.f. following subsections) here also depend on the discrete model of biological cells in the sense that the direction of deformation jumps at these boundaries (c.f., Fig. 6a). These discontinuities represent the fact that the cytoskeleton is pulling from both directions at the boundary regions between biological cells. However, the overall deformation, **F**=**F**_*e*_**F**_*a*_, includes also the simultaneous passive (elastic) response, maintaining (among others) the continuity of the tissue. In terms of biophysics, the discrete part of our model represents the cytoskeleton associated with the plasma membrane of each biological cell (actomyosin cortex), where active deformations may be driven by myosin motor proteins. In contrast, the continuous model part represents the body of the cell lumina.

### Deformation gradient decomposition and model equations

In finite strain theory, structural dynamics are expressed in the Lagrangian or particle-centered framework (as opposed to the Eulerian framework where a fixed point **x** in space is observed): Let **X** be a particle in the undeformed configuration and **x**=**x**(**X**,*t*) be its current position at time *t*. Then, the vector **u**(**X**,*t*)=**x**(*X,t*)−**X** joining these positions is called displacement and the deformation and its gradient are defined as:

$${} \mathbf{T} \colon\! \langle \mathbf{X},t \rangle \mapsto \mathbf{X} + \mathbf{u}(\mathbf{X},t), \quad\! \mathbf{F} \colon\! \,=\, \nabla \mathbf{T} =\! \nabla \mathbf{u} + I,\!\!\quad J = \det(\mathbf{F}). $$

Local deformation processes will be incorporated using the multiplicative deformation gradient decomposition [47], splitting the deformation gradient into two parts:

(1) $$ \mathbf{F} = \mathbf{F}_{e} \mathbf{F}_{a}(c),  $$

where **F**_*a*_(*c*) is a prescribed active deformation depending on the morphogen concentration *c* and **F**_*e*_ is the passive elastic response to these active changes, which ensures the continuity of the overall deformation **F** and minimizes the mechanical stress (see also Fig. 6a). Numerically, this means to replace **F**_*e*_ by $\mathbf {F} \mathbf {F}_{a}^{-1}$ and to solve for the overall deformation **F**, applied to the continuous tissue body. Thus, although **F**_*a*_ may show discontinuities at boundary regions between biological cells, the smoothness of the overall deformation is naturally given by the interplay between active and passive processes leading to the smooth finite element solution determined by **F**.

Furthermore, the active deformation **F**_*a*_ (influencing the shape of the biological cells, c.f. Fig. 6a) is directly determined by morphogen concentrations *c*. This is a common assumption in the mechanochemial modeling of tissue morphogenesis [1, 36, 43], and is based on the idea that morphogens locally induce remodeling of the cytoskeleton, which again determines the cell shape. In accordance, various experimental results show that different active deformation processes (such as cell-shape changes or local tissue growth) can be directly induced by expression of signaling molecules [9, 27, 35, 54]. However, the final tissue shape is not entirely determined by **F**_*a*_(*c*) but rather by the interplay between **F**_*a*_(*c*) and the elastic response **F**_*e*_ (c.f., Fig. 6a). In this way, local morphogen concentrations (respectively the resulting **F**_*a*_(*c*)) can induce complex patterns of stress, strain, and compression within the surrounding tissue (Fig. 6b).

The shape of structural equations is determined by the two principles of conservation of mass and momentum. In Eulerian coordinates they read:

$$\begin{aligned} \rho &= J \rho^{0}, \qquad\qquad\text{as well as}\\ \rho \partial_{tt} \mathbf{u} &= \rho \mathbf{f} +\text{div}(\mathbf{\sigma}_{e}), \end{aligned} $$

for current and initial mass distributions *ρ*(**x**,*t*),*ρ*^0^(**x**), external forces **f** and surface forces **σ**_*e*_. The divergence comes up as their surface integral is transformed to the volume by Gauss’ integration theorem. Finally, the equation is transformed to the Lagrangian framework via the Piola transform and the relation $\mathbf {F}_{e}\mathbf {\Sigma }_{e} = J_{e} \mathbf {\sigma }_{e} \mathbf {F}_{e}^{-T}$.We use the simple nonlinear St. Venant-Kirchhoff model for compressible, hyperelastic materials to model the tissue. It models the relation between the second Piola-Kirchhoff stress tensor **Σ**_*e*_ and the Green-Lagrangian strain tensor **E**. Eventually, it is coupled with a reaction-diffusion equation which accounts for modeling the morphogen concentration by

(2) $$ \partial_{t} c - \text{div}\left(D \nabla c\right)- R = 0,  $$

which is transformed to the reference framework using the divergence of the Piola transformation. Thus, the final system reads: Find displacement **u** and morphogen concentration *c* such that

(3) $$ \begin{aligned} \epsilon \rho^{0} \partial_{tt} \mathbf{u}-\text{div}\left(\mathbf{F}_{e}\mathbf{\Sigma}_{e}\right) &= \rho^{0} \mathbf{f} \quad \text{and}\\ J_{e} \partial_{t} c - \text{div}\left(J_{e} \left(\mathbf{F}_{e}^{t} \mathbf{F}_{e}\right)^{-1} D \nabla c\right)- J_{e} R &= 0 \end{aligned}  $$

hold, where

(4) $$ {}\mathbf{\Sigma} \,=\, \lambda \operatorname{tr}(\mathbf{E}_{e}) \mathbf{I} + 2 \mu \mathbf{E}_{e},\!\!\! \quad \mathbf{E}_{e}\! =\! 0.5\! \left(\!\mathbf{F}_{e}^{T}\mathbf{F}_{e}\!- \!\mathbf{I}\!\right),\, \text{and} \quad \mathbf{F}_{e} \,=\, \mathbf{F} \mathbf{F}_{a}^{-1},  $$

with the Lamé constants *μ* and *λ*. The reaction term *R*=*R*(**Σ**_*e*_,**E**_*e*_,**F**_*e*_,*c*) will incorporate the feedback of the mechanics on the morphogen level. With the exception of the second predictive *in silicio* experiment which is presented further on, the right hand side is set to **f**=0, i.e. external forces are usually not considered.

We emphasize that we are interested in a quasi-stationary state where the time derivative in the structural equation is not considered. In practice however, the time derivative multiplied by *ε*=0.1 is used for stabilization of the numerical scheme. The specific choice of *ε* does not significantly change the final pattern. Furthermore, the evolution equation with non-zero time derivative yields uniqueness of the numerical solutions, since homogeneous Neumann boundary conditions are assumed (for more details, we refer to the section “Finite element (FEM) approximation”). Consequently, the solution of the quasi-stationary system is unique up to translations and rigid body rotations. Such solutions might affect the convergence of the numerical scheme. Therefore, we consider a small parameter *ε* to balance the oscillatory behavior introduced by the time derivative with rigid body rotations experienced in the quasi-stationary setup. With such stabilization term, oscillations are largely over-damped with a negligible loss of convergence speed.

### Mechanochemical events

**Active deformations** Mechanochemical pattern formation requires an interplay between active and passive chemical and mechanical processes. Especially, the morphogen concentration *c* couples into the structural equation through the active part of the decomposed deformation gradient (Eq. (1)).

In this study, we focus on an active deformation process called an apical or a basal constriction, since this is a common deformation process during tissue morphogenesis [34, 38, 50]. Mathematically, this kind of deformation can be expressed by the active part of the deformation gradient tensor. Discrete biological cells are now introduced into a continuum-based model by a piecewise description of the active deformation gradient **F**_*a*_. Each biological cell is given a “material id” to distinguish between different biological cells, and is furthermore represented by 64 finite elements. For a schematic view of the deformation gradient decomposition with apical constriction of discrete biological cells we refer to Fig. 6. To define the active deformation tensor **F**_*a*_, we first introduce local coordinate systems $\hat {\mathbf {X}}$ in the origin of every cell, oriented such, that $\hat {\mathbf {X}}_{1}$ points in the radial direction. By $\mathbf {T} = \frac {\partial \hat {\mathbf {X}}}{\partial {\mathbf {x}}}$ we denote the mapping from **x** to these parametric coordinates. Here, the constriction tensor is defined as

(5) $$ \hat{\mathbf{F}}_{a} := \left(\begin{array}{cc} 1 + k c \hat{\mathbf{X}}_{1} & k c \hat{\mathbf{X}}_{0} \\ 0 & 1 \end{array}\right),  $$

where *k* is a constant and $\left (\hat {\mathbf {X}}_{0},\hat {\mathbf {X}}_{1}\right)$ are the 2D coordinates in the cell-wise reference system. For positive values of *k*, this results in apical constriction and for negative values in a basal one.

The complete active deformation tensor is combined given as

(6) $$ \mathbf{F}_{a} = \mathbf{T}^{-1} \hat{\mathbf{F}}_{a} \mathbf{T}.  $$

We point out that the elastic Green-Lagrangian strain tensor **E**_*e*_ in Eq. 4 and subsequently the Cauchy stress tensor *σ*_*e*_ remain symmetric, since

$${} \mathbf{E}_{e}^{T} = \frac{1}{2} \left(\mathbf{F}_{a}^{-T}\mathbf{F}^{T}\mathbf{F}\mathbf{F}_{a}^{-1} - \mathbf{I} \right)^{T} = \frac{1}{2} \left(\left(\mathbf{F}_{a}^{-T}\mathbf{F}^{T}\mathbf{F}\mathbf{F}_{a}^{-1}\right) - \mathbf{I} \right) = \mathbf{E}_{e} $$

holds. Furthermore, the active deformation **F**_*a*_ is volume-preserving which can be seen for the volume *V* of a single biological cell by

$$\begin{array}{*{20}l} V_{a} &= \int_{{V}_{a}} \mathrm{d} \mathbf{X}_{1} \mathrm{d} \mathbf{X}_{0} = \int_{V} |\det(\hat{\mathbf{F}}_{a})| \mathrm{d} \hat{\mathbf{X}}_{1} \mathrm{d} \hat{\mathbf{X}}_{0} \\ &= \int_{V} (1+k c \hat{\mathbf{X}}_{1}) \mathrm{d} \hat{\mathbf{X}}_{1} \mathrm{d} \hat{\mathbf{X}}_{0} = V + k c \underbrace{\int_{V}\hat{\mathbf{X}}_{1} \mathrm{d} \hat{\mathbf{X}}_{1} \mathrm{d} \hat{\mathbf{X}}_{0}}_{=0}, \end{array} $$

where the last integral vanishes since integration over **x**_1_ cancels out.

**Mechanotransduction** The role of mechanosensitive mechanisms controlling chemical cellular processes has been extensively studied within the last decade [12, 30]. Different types of mechanical cues have been shown to influence gene expression (such as morphogen production), namely stress [28, 41, 42], compression/stretch [5] but also geometrical constraints determining the strain/cell-shape [29, 56]. In terms of continuum mechanics, these three cues can be expressed via invariants of the corresponding tensors. Tensor invariants were chosen since their values do not change with the rotation of the coordinate system, which is equivalent to a rotation of the initial morphogen concentration. Thus, using tensor invariants, the feedback and consequently the solution of our system will rotate accordingly to how the initial conditions were rotated.

Since there are two tensor invariants available in 2D, there are a total of six possible candidates resulting from mechanical stress, compression/stretch and strain.

**Fig. 5:**
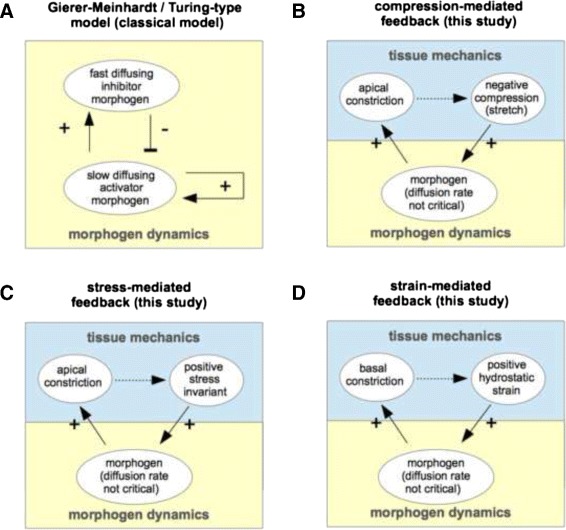
Proposed interactions in the context of different pattern formation models. **a** the Turing-type Gierer-Meinhardt model, **b**–**d** the three types of mechanochemical feedback loops which are the examples considered in the present study. Continuous arrows indicate explicit model assumptions, dotted arrows depict passive mechanical relationships

**Appropriate feedback loops** To obtain mechanochemical feedback loops leading to *de novo* pattern formation, we combine morphogen dynamics with experimentally observed active strains and mechanotransduction processes as motivated above. Appropriate feedback loops leading to such pattern formation have been received by extensive simulation studies of possible candidates, keeping the general “long-range inhibition/short range activation” principle in mind. Here, we exemplarily present three different feedback loops (c.f., Figs. 1 and 5b–d), each representing one certain type of mechanotransduction. Thus, each feedback loop uses a tensor invariant *I*, based on a tensor representing one of the the three different mechanical cues mentioned above, namely

1. the determinant of the deformation gradient *I*= det(**F**), which has the physical interpretation of compression or stretch. More precisely, $\det (\mathbf {F}) = \frac {V_{a}}{V_{0}}$ is the ratio of the deformed to the initial volume;
2. the isotropic strain, which is the trace of the Green-Lagrangian strain tensor, i.e., *I*= tr(**E**). This measure represents the hydrostatic strain which is the displacement between particles in the principal coordinate direction inside the tissue relative to a reference length; and
3. the determinant of the elastic Cauchy tress tensor $I = \det (\mathbf {\sigma }_{e}) = \det \left (J_{e}^{-1}\mathbf {F}_{e} \mathbf {\Sigma }_{e} \mathbf {F}_{e}^{T}\right)$. Let us note that **σ**_*e*_ is used since the determinant of the antisymmetric tensor **Σ**_*e*_ is not an invariant. **σ**_*e*_ expresses the stress which biological cells are experiencing as a result of their active deformation.

**Fig. 6:**
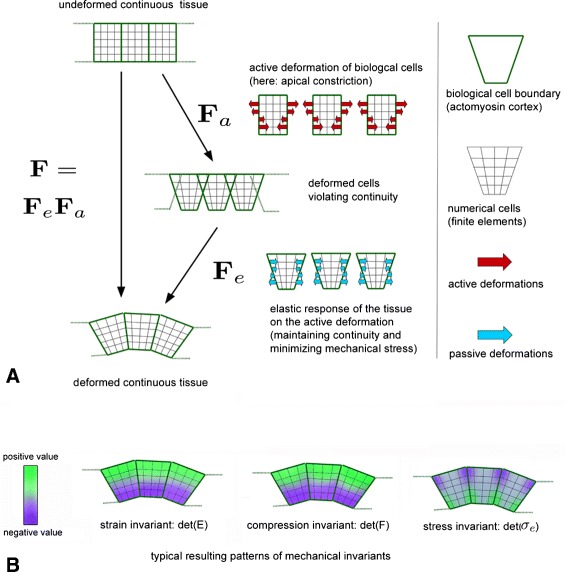
Scheme of the modeling approach. **a** The interplay between active deformations (applied to biological cells) and the passive response of the tissue on these active deformations in order to maintain continuity and to minimize the mechanical stress. **b** Typical patterns of the three different mechanical invariants as a result of a local active deformation

Conceptually, stress is an internal force acting on a boundary per unit area of this boundary, while strain or compression are measures of deformation. For an illustration of the qualitative differences between these three different tensor invariants, we refer to Fig. 6b. It appears that differences between compression and the strain invariant are usually small and are apparent only at the boundaries of biological cells.

In the following, we term the three corresponding feedback loops as “compression-mediated feedback” (1), "strain-mediated feedback” (2) and “stress-mediated feedback” (3), respectively.

The feedback of mechanics on morphogen dynamics is incorporated by the reaction term *R*, as stated in Eq. (2). Thereby, the tensor invariants *I* are included via the Michaelis-Menten kinetics by

(7) $$ R = k_{2} \frac{\max\{I,0\}}{k_{m} + \max\{I,0\}} - k_{1} c,  $$

with positive constants *k*_1_,*k*_2_,*k*_*m*_>0. Here, *k*_1_ is equal for all three considered feedback loops and causes a constant morphogen degradation in the entire tissue. The first term on the right hand side of Eq. (7) represents the impact of mechanics on the morphogen production: If the value of the respective tensor invariant *I* is positive, morphogen is produced. Else, no feedback is generated. For large values, this term converges towards *k*_2_ which can consequently be interpreted as the maximal morphogen production rate. *k*_*m*_ is the Michaelis constant and is the value of *I* where half of the maximal production rate *k*_2_ is reached.

Michaelis-Menten kinetics has been used to reflect the fact that there exists a maximal expression rate of the morphogen promoter. Thus, morphogen production saturates for large values of *I*, i.e., for large deformations, stresses, or strains.

Finally, we combine the above mentioned active deformations and mechanotransduction processes to simple positive feedback loops by the following two assumptions (c.f., Fig. 5b–d):

- local morphogen accumulation leads to local tissue deformations (in terms of apical or basal constriction), and
- different mechanical cues (such as compression, strain, or stress) can induce the production of this morphogen in turn.

### Predictive *in silicio* experiments

To demonstrate a possible experimental outcome indicating the existence of a mechanochemical feedback within a tissue, we have performed two predictive *in silicio* experiments using the strain-mediated feedback loop as an example. For the detailed motivation of the following two experiments we kindly refer the reader to the Results and discussion section.

First, to mimic experimental and local ectopic morphogen production, we have added a constant production term to Eq. (2) within a certain subset *Ω*_*s*_⊂*Ω*. In particular, the equation now reads

$$\partial_{t} c - \text{div}\left(D \nabla c\right) = \left\{ \begin{array}{ll} R + (1-\tfrac{c}{c_{max}})c & \text{if}\, (r,\phi) \in \Omega_{s} \\ R & \text{else}, \end{array}\right. $$

where *c*_*max*_ was set to 9·10^−9^*molm*^−2^, since this was about the highest concentration which was reached for the unmodified strain-mediated feedback loop and thus represents the natural maximal expression rate. $\Omega _{s} := \{(r,\phi) \in \mathbb {R}^{\geq 0} \times \mathbb {R} \mid 135 \,\mu m \leq r \leq 150 \,\mu m, \frac {11}{8} \pi \leq \phi < \frac {13}{8} \pi \}$ is a circular sector in the polar coordinates, compare also Fig. 3a.

Second, to simulate a prescribed external deformation, we made the specific choice of an outward-pulling external force as the right hand side of the structural Eq. (3) in our system,

$$\epsilon \rho^{0} \partial_{tt} \mathbf{u} -\text{div}\left(\mathbf{F}_{e}\mathbf{\Sigma}_{e}\right) = \rho^{0} \mathbf{f}. $$

by defining **f**=(**f**_0_,**f**_1_)^*T*^ as

$$\mathbf{f}_{0} := 0, \quad \mathbf{f}_{1} := \left\{ \begin{array}{ll} - 0.5 \cdot e^{-k_{x} {x_{0}^{2}}}e^{-0.00025 t} & \text{if}\,\, \mathbf{x}_{1} < 0 \\ 0 & \text{else}, \end{array}\right. $$

with *k*_*x*_=9·10^9^. The first component **f**_0_ of this volume force is always zero. Since the center of the tissue is in the origin, the second component, **f**_1_, constitutes a pull in negative **x**_1_ direction, i.e., downwards. The pull is localized around **x**_0_=0 where it is strongest and decreases exponentially for larger absolute values of *x*_0_; see also Fig. 3c. Note that **x**_1_<0 excludes a second pull downwards on the top of the tissue. Also, it is strongest in at the beginning of the deformation process and decreases exponentially in time.

### Finite element (FEM) approximation

For discretization in space, different feedback loops required different levels of mesh-refinement. On the one hand, for the compression- and strain-mediated ones, the entire domain *Ω* has been split into 4096 finite elements, see Fig. 4a. Here, each biological cell is thus represented by 64 finite elements. Especially, isoparametric *Q*1 finite elements have been used (where *Q*1 depicts an approximation with polynomial ansatz functions of degree one).

On the other hand, the stress-mediated feedback loop depends not only on the smooth solution *u* but also on the non-smooth measure $\mathbf {F}_{a}^{-1}$, which required a mesh of at least 262144 cells in order to properly resolve the stress-mediated feedback within the *Q*1 ansatz. It turned out that shorter computation times were possible using *Q*2 finite elements instead, i.e., with polynomial ansatz functions of degree two. This approach, combined with local mesh refinement, reduced the total amount of cells to a total of 17944.

Discretization in time has been done using a simple Theta-Time-Stepping method. Finally, for all simulations we have used homogeneous Neumann boundary values

$$ \mathbf{F}_{e} \mathbf{\Sigma}_{e} \mathbf{n} = 0 \quad \text{on} \partial \Omega, $$

where **n** is the unit vector in normal direction on the boundary. It thus prescribes that the deformation gradient times the Kirchhoff stress in normal direction on the boundary is zero. All simulations are done using the software library GASCOIGNE 3D [3] and its parallelized multigrid method.

### Parameter setup

Values of the Lamé constants are usually given in terms of the Young’s modulus *E* and Poisson’s ratio *ν*. They can be obtained by the conversion formulas

$$\mu = \frac{E}{2(1+\nu)}\, \text{and}\, \lambda = \frac{E\nu}{(1+\nu)(1-2\nu)}. $$

For the following calculations we have used *E*=100*Pa* and *ν*=0.4, similar to the assumptions made in [8], and *ρ*^0^=1000*kgm*^−2^ for the initial mass distribution. Furthermore, we have always set: *D*∼10^−14^*m*^2^*s*^−1^ for the diffusion coefficient, *k*_1_∼10^−4^*s*^−1^ for the degradation rate of the morphogen level in the entire domain and *k*_2_∼10^7^*molm*^−2^*s*^−1^ for the maximal morphogen production rate. The Michaelis constant was set to *k*_*m*_=0.1 which means that for positive feedback *I*=*k*_*m*_ half of the production rate *k*_2_ is reached.

Finally, uniformly distributed random concentrations for each biological cells or a morphogen gradient were used as initial conditions. In both cases, the morphogen concentration was prescribed in the interval *c*∈[0,10^9^]*molm*^−2^. For visualization in the following results, initial conditions were transformed into the interval *c*∈[0,1]*molm*^−2^. The scale of the morphogen does not influence results, since only the constant *k* which determines how strong the morphogen concentration couples into the active deformation gradient (see Eq. (5)), has to scale in the same manner. It was set to *k*∼10^−6^*mol*^−1^*m*.

## Author’s response

### Revision 1

#### Reviewer 1: Marek Kimmel – Rice University, Houston, Texas

**Summary:**

The manuscript presents a novel explanation of the morphogen in pattern formation mechanisms in development. It is proposed that since the putative purely chemical morphogens have been difficult to find, it is necessary to look for other possibilities. One such possibility is offered by mechanochemistry. Authors carefully demonstrate, using a mathematical model, how tissue mechanics provides a signaling modality. This is a clearly written and interesting paper.

**Suggestions:**

1. I like the way the paper is written. The only suggestion I may have is that since the readership of Biology Direct may be somewhat less familiar with specialized models of pattern formation, the authors provide a descriptive, mathematical, or graphical representation of the "classical" Gierer and Meinhardt (or similar) model, so that the failure to find the putative morphogen is explained using a more specific example.
  *We have added a more detailed mathematical description of the classical Gierer-Meinhardt model as well as corresponding equations to the manuscript (page 2, paragraph 1-2). Furthermore, we have added a a graphical representation of these models to Fig.**5**(c.f., Fig.**5**a*).

#### Reviewer 2: Konstantin Doubrovinski (nominated by Ned Wingreen) – Universität des Saarlandes, Saarbrücken, Germany

**Summary:**

In their manuscript the authors present a mathematical model of morphogenesis that involves mechanosensitivity, i.e. a model where the dynamics of morphogen gradients depends on stress distribution in the tissue. It has long been known that biochemical processes can influence tissue mechanics, for example through control of molecular motors. In the recent years it is becoming increasingly realized that mechanical stresses can also influence biochemistry. In particular, it has been experimentally demonstrated that cell division rate and planar cell polarity in certain tissues can be influenced by external forces applied to those tissues. The model proposed by the authors is concisely summarized on page 8. The corresponding equations express conservation of momentum in the tissue and conservation of mass of the morphogen. Morphogen concentration drives tissue deformation through a contribution to the active deformation gradient (Eq. 3). Mechanosensitivity enters the description through a source term in the equation that describes morphogen dynamics. This source is assumed to be a function of some invariant of stress (or strain) tensor. The authors consider three models that incorporate three possible scalar invariants that might influence morphogen dynamics (listed on page 9). The paper discusses a subject of significant interest to biology community and in my opinion is appropriate for the intended readership.

On the technical side, there are a number of points that I think require clarification.

**Suggestions:**

1. In the equation proposed by the authors the active deformation is directly determined by morphogen concentration. To me it appears more natural to assume that stress (rather than deformation) is a function of concentration. Could the authors comment on this?
  *We have added a corresponding paragraph including a discussion of this topic as well as additional references to the manuscript (c.f., page 9).*
2. It seems to me that active deformation (denoted by *F*_*a*_) is a tensor and should thus transform like one (just like scalar invariants describing the coupling of stress and morphogen concentration dynamics transform like scalars). Equation 3, however, does not appear an isotropic tensor to me. I would rather expect this tensor to be a scalar multiple of identity matrix, where the scalar factor may depend on morphogen concentration. In any case, stress must be a symmetric tensor. The authors must explicitly show that their particular choice of *F*_*a*_ ensures that stress transforms as a tensor and is symmetric.
  *The deformation gradient***F**_*a*_*itself must not be symmetric or isotropic, it is just the gradient of any active mapping. Considering isotropic growth,***F**_*a*_=*cIwould indeed be the correct choice. We have added on page 11 a section explaining the setup of***F**_*a*_*in the case of basal or apical constriction in detail. Further, we have shown, that the elastic Green-Lagrangian strain tensor***E***in Eq.* (4) *is always symmetric, c.f. page 11. Hence, every stress tensor derived from***E***will be symmetrix.*
3. In an actual biological setting I would expect the dynamics to be strongly overdamped implying that the dv/dt term in momentum balance equation must vanish. Was this true of the simulated regime?
  *We consider a quasi-stationary system by considering dv/dt only as a stabilization term, providing uniqueness of solutions. We have clarified this by adding a corresponding paragraph to the manuscript (c.f., page 10, last paragraph, as well as Eq.* (3)). *Actual biological settings will be damped by various factors, that are not present in our simplified models.*
4. The section on the details of *in-silicio* simulation is much too short to fully appreciate the details of the implementation. I think this section must be substantially extended.
  *As suggested by the referee, we have substantially extended this section (c.f., page 13-14).*

#### Reviewer 3: Jun Allard (nominated by William Hlavacek) – University of California, Irvine

**Summary:**

Morphogenesis, the process by which a tissue obtains its shape during development, is driven by both diffusing factors and mechanical cues, which effect morphogenesis by influencing both growth and mechanical deformation. All of these mechanisms have experimental support and have been included in mathematical models. This manuscript explores morphogenesis by mechanical cues acting on mechanical deformation of the tissue using a mathematical model. Specifically, the authors simulate a thin loop of tissue confined to a two-dimensional plane. The tissue is assumed to be hyper-elastic and experiences an active internal stress, which depends on a diffusing factor, whose production in turn depends on local mechanics (strain or stress). The authors investigate what shape the loop takes. My two main concerns are

- clarity in describing their model, especially its geometry, and
- the generality of their results. In addition,
- the conceptual distinction between the three mechanical invariants should also be clarified.

**Suggestions:**

1. The authors should edit the text to clarify that this is a thin loop of tissue confined to the plane. This is important because the restricted geometry limits the significance of the results. - Add a paragraph at the beginning of “Results and discussion” stating the model geometry. Two or three sentences would be sufficient. - Delete phrases like “tissue sphere” (p.3), which risk being misleading. - Change the dimensionality language, for example at the top of p.4. The tissue is quasi-1D in a 2D domain, so describing it as 2D is ambiguous. The sentence “in 2D domains there is no difference between stripes and spots” should be changed to something like, “In a thin strip, there is no difference between stripes and spots”. Figure 4 also risks being misleading, since the model is strictly continuum-based, and at no point are “biological cells” employed in the model. Indeed, Fig. 4 could be removed completely.
  *As suggested by the referee, we have revised the entire manuscript regarding the dimensionality language (see for example page 5, second paragraph), we have added additional information to the beginning of the* “*Results and discussion*” *section (page 3, last paragraph), and have added information to the* “*Model geometry*” *subsection at the beginning of the* “*Methods*” *section (page 8, fist paragraph).*
  *Biological cells play an important role in our modeling approach, since our approach is not strictly continuum based but combines continuous finite strain models with discrete cellular approaches. To clarify our approach, we have changed the following points correspondingly in order to make it clearer:*
  *We have added an additional Figure (Fig.**6**a*) *to the manuscript where we graphically present the relationship of biological versus numerical cells;*
  *we have added a subsection to the manuscript* (“*Combining continuous and discrete tissue models*”), *where we provide detailed information (page 8);*
  *we have added additional information to the “Active deformations” subsection (page 11); and*
  *we have added a corresponding sentence to the abstract.*
2. The model (like all mathematical models) chooses specific functional forms for, e.g., diffusing factor production and active force response to the diffusing factor. What results do the authors think are general, and robust to changes in the specific form of Eq. 3 or Eq. 4? What specific qualitative (or quantitative) predictions are made? For example, do the authors propose that, in general, strain-dependent feedback does not give gastrulation-like invaginations, while stress-mediated feedback does? Do the authors propose that, in general, thinner tissues will have more bumps, as in Fig. 2? In the absence of general conclusions, it is difficult to evaluate the impact of this work.
  *We are a little bit cautious with general conclusions, since we know that patterns resulting from simulation of tissues restricted to the 2D plane may distinctly differ from those of full 3D tissues, for several reasons (examples are give with the second paragraph, page 5). However, we have added information regarding the robustness ob observed results at several places within the manuscript, e.g. page 4 paragraph 2 (robustness regarding initial conditions), page 4 paragraph 3 (robustness regarding the type of active deformations), page 5, last sentences (robustness regarding the mechanical invariant used within the feedback), and page 5, first paragraph (non-robustness of pattern symmetry). Finally, we have added a corresponding sentence to the**Conclusions**section (page 7).*
3. It is difficult to intuitively understand the three mechanical invariants (strain, compression and stress). The authors should add heat maps showing the three invariants somewhere. For example, the twelve configurations in Fig. 1 could be accompanied by duplicates with heat maps showing the invariant used. It would also greatly enhance the accessibility of the paper if the authors could add a conceptual description of the difference between strain, compression and stress in the text.
  *In order to address this point, we have added the following information to the manuscript:*
  *We have added schematic graphical examples of the different types of mechanical invariants (Fig.**6**b*)
  *We have added corresponding heat maps to Fig.**1**(right-hand side).*
  *We have added a conceptual description of these invariants to the manuscript (Page 12).*
4. Tissue growth plays an important role in morphogenesis, especially in embryogenesis. The authors should state in the text that they are neglecting tissue growth.
  *We have added a corresponding statement to the manuscript (page 4, paragraph 3).*
5. It is unusual that the authors consider inertia in their model, because biological processes at this scale are in the low-Reynolds, non-inertial regime. The authors should state that the results do not depend on mass-density or the inertia terms, or note if they do. (Is there a separation of timescales between extremely fast mechanics and slow diffusion, production and degradation of the diffusing factor?)
  *This question is related to remark 3 of referee 2 (Konstantin Doubrovinski). Inertial effects do not play a role in this model. However, instead of completely removing this term and solving for the stationary limit, we add the time derivative for reasons of stability. Temporal scales however are separated, which is realized by the parameterεin Eq.* (*3*). *Numerically, the specific choice ofεdoes not change the final pattern.*
6. Inconsistency: The main text says Fig. 2 is compression-mediated, while the caption says it is strain-mediated.
  *We have changed this*
7. There are several grammatical and spelling errors. For example, on p.11 Line 16, change “I turned out that?” to “It turned out that?” There is also some language usage that is unusually colloquially, notably: “Anyway, the scale of the morphogen is not crucial, since?”, suggest change to “The scale of the morphogen does not influence results, since?”
  *We have changed this*

### Revision 2

#### Reviewer 1: Marek Kimmel – Rice University, Houston, Texas

No additional comments on the manuscript.

#### Reviewer 2: Konstantin Doubrovinski (nominated by Ned Wingreen) – Universität des Saarlandes, Saarbrücken, Germany

The authors have adequately addressed my previous concerns.

1. I believe in the text following reaction-diffusion equations diffusion constants initially denoted with *d*’s are (erroneously?) referred to by *μ*’s.
  *We have changed this.*

#### Reviewer 3: Jun Allard (nominated by William Hlavacek) – University of California, Irvine

1. The manuscript is much improved and much clearer in its description of the model. p.4 I suggest not using the term "deformation" to include tissue growth. Therefore, I suggest changing "However, other possible active deformations" to "However, other possible active processes". *We have changed this.*
2. From this version, it appears that the authors are making the assumption that active deformation only occurs at the boundaries of biological cells. This assumption is reasonable but could be more clearly stated? in its current writing, it?s easy to miss. I suggest adding a sentence on p.8 saying explicitly that active forces are only applied at the boundaries of biological cells, and again near Eq. 1 (on p.9).
  *Actually this is not the case, we apologize that our description was misleading. The active deformation is applied to the whole tissue body rather than only at the cell boundaries. However, the biological cell boundaries play indeed a very special role during this process, since the direction of deformation shows a jump at these boundaries. This is a result of the cytoskeleton pulling from both directions at the boundary region, the latter separating biological cells. To clarify this, we have added/changed corresponding sentences at page 8 and page 9, as suggested by the referee, and hope that this is clearer now.*
3. Minor corrections: pg 2, second sentence of background - “pattering” change to “patterning” pg 3, last sentence - “bophysical” change to “biophysical” pg 8, second to last sentence - “cytosceleton” change to “cytoskeleton”
  *We have corrected this.*

## Abbreviations

2D: two dimensional
3D: three dimensional
FEM: finite element method
